# Supplementary material for: Synthesis of Hydrophobic Nanosized Silicon Dioxide with a Spherical Particle Shape and Its Application in Fire-Extinguishing Powder Compositions Based on Struvite
Source: Nanomaterials (Basel). 2023 Mar 27;13(7):1186. doi: 10.3390/nano13071186 (PMC10096559; doi:10.3390/nano13071186)
Supplement: Supplementary file 1 [file nanomaterials-13-01186-s001.zip › nanomaterials-2292981-supplementary.pdf]

Supporting information

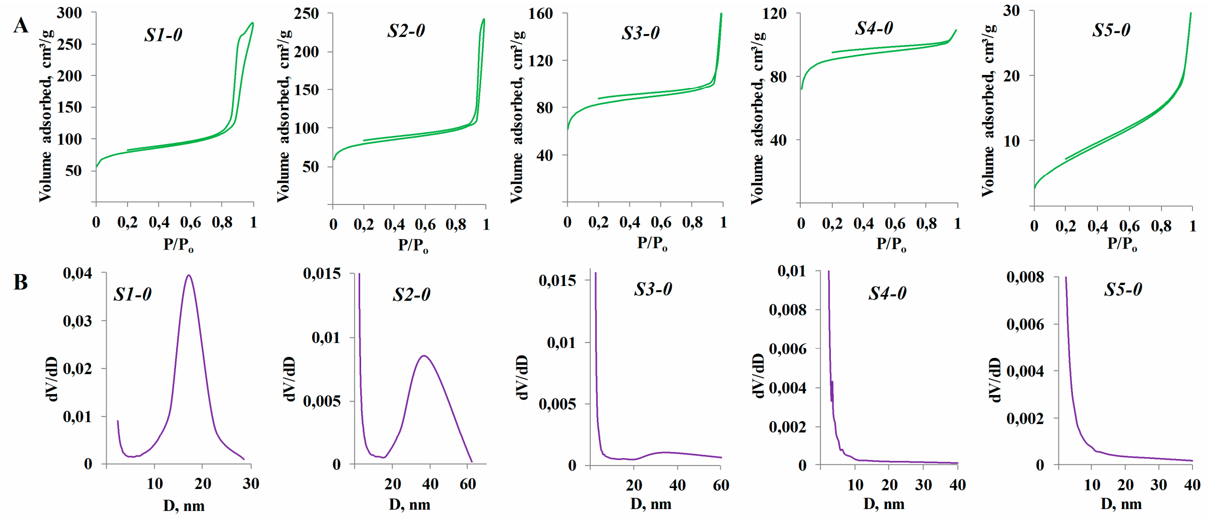

Figure S1. Sorption-desorption isotherms (**A**); pore size distribution in the initial SiO_2 samples, with differet [H2O]/[TEOS] ratio (**B**).

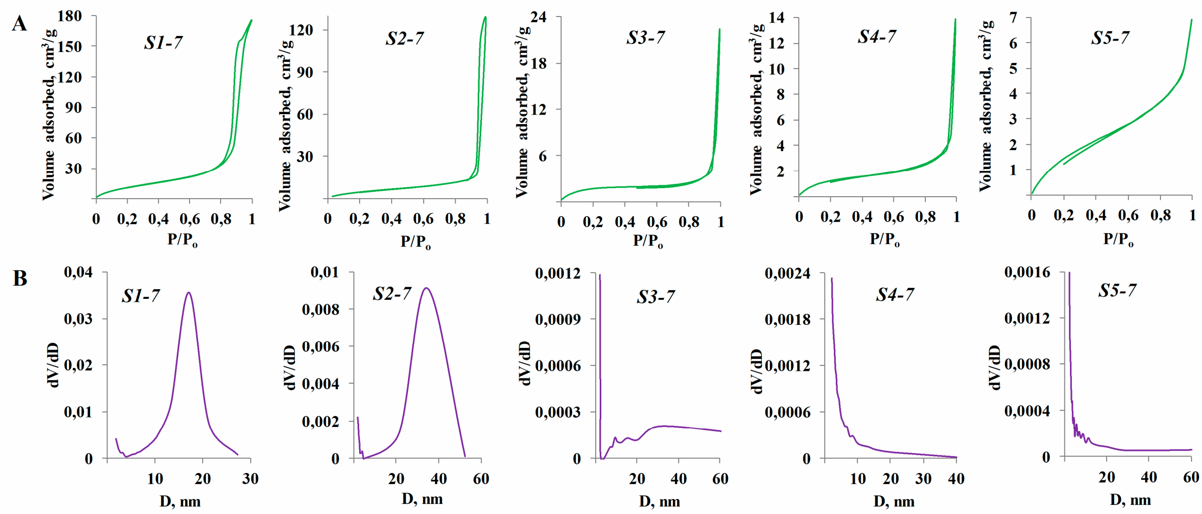

Figure S2. Sorption-desorption isotherms (**A**); pore size distribution in SiO_2 samples hydrophobized with 7% PMHS and with different particle sizes (**B**).
